# Whole intestinal microbiota transplantation is more effective than fecal microbiota transplantation in reducing the susceptibility of DSS-induced germ-free mice colitis

**DOI:** 10.3389/fimmu.2023.1143526

**Published:** 2023-05-09

**Authors:** Yapeng Yang, Jinhui He, Yuqing Wang, Lifeng Liang, Zeyue Zhang, Xiang Tan, Shiyu Tao, Zhifeng Wu, Miaomiao Dong, Jixia Zheng, Hang Zhang, Shuaifei Feng, Wei Cheng, Qiyi Chen, Hong Wei

**Affiliations:** ^1^ Central Laboratory, Clinical Medicine Scientific and Technical Innovation Park, Shanghai Tenth People’s Hospital, Tongji University, Shanghai, China; ^2^ State Key Laboratory of Agricultural Microbiology, College of Animal Sciences and Technology, Huazhong Agricultural University, Wuhan, Hubei, China; ^3^ Precision Medicine Institute, The First Affiliated Hospital, Sun Yat-sen University, Guangzhou, China; ^4^ Intestinal Microenvironment Treatment Center, Tenth People’s Hospital of Tongji University, Shanghai, China

**Keywords:** whole intestinal microbiota transplantation, fecal microbiota transplantation, inflammatory bowel disease, germ-free mice, 16S rDNA

## Abstract

Fecal microbiota transplantation (FMT) is an emerging and effective therapy for the treatment of inflammatory bowel disease (IBD). Previous studies have reported that compared with FMT, whole intestinal microbiota transplantation (WIMT) can more precisely replicate the community structure and reduce the inflammatory response of the host. However, it remains unclear whether WIMT is more effective in alleviating IBD. To examine the efficacy of WIMT and FMT in the intervention of IBD, GF (Germ-free) BALB/c mice were pre-colonized with whole intestinal microbiota or fecal microbiota before being treated with dextran sodium sulfate (DSS). As expected, the symptoms of colitis were alleviated by both WIMT and FMT, as demonstrated by the prevention of body weight loss and decreased the Disease activity index and histological scores in mice. However, WIMT’s anti-inflammatory effect was superior to that of FMT. In addition, the inflammatory markers myeloperoxidase (MPO) and eosinophil peroxidase were dramatically downregulated by WIMT and FMT. Furthermore, the use of two different types of donors facilitated the regulation of cytokine homeostasis in colitis mice; the level of the pro-inflammatory cytokine IL-1β in the WIMT group was significantly lower than that in the FMT group, while the level of the anti-inflammatory factor IL-10 was significantly higher than that in the FMT group. Both groups showed enhanced expression of occludin to protect the intestinal barrier in comparison with the DSS group, and the WIMT group demonstrated considerably increased levels of ZO-1. The sequencing results showed that the WIMT group was highly enriched in *Bifidobacterium*, whereas the FMT group was significantly enriched in *Lactobacillus* and *Ochrobactrum*. Correlation analysis revealed that *Bifidobacterium* was negatively correlated with TNF-α, whereas *Ochrobactrum* was positively correlated with MPO and negatively correlated with IL-10, which might be related to different efficacies. Functional prediction using PICRUSt2 revealed that the FMT group was considerably enriched in the L-arginine biosynthesis I and L-arginine biosynthesis IV pathway, whereas the WIMT group was enriched in the L-lysine fermentation to acetate and butanoate pathway. In conclusion, the symptoms of colitis were subsided to varying degrees by the two different types of donors, with the WIMT group being more effective than the FMT group. This study provides new information on clinical interventions for IBD.

## Graphical Abstract

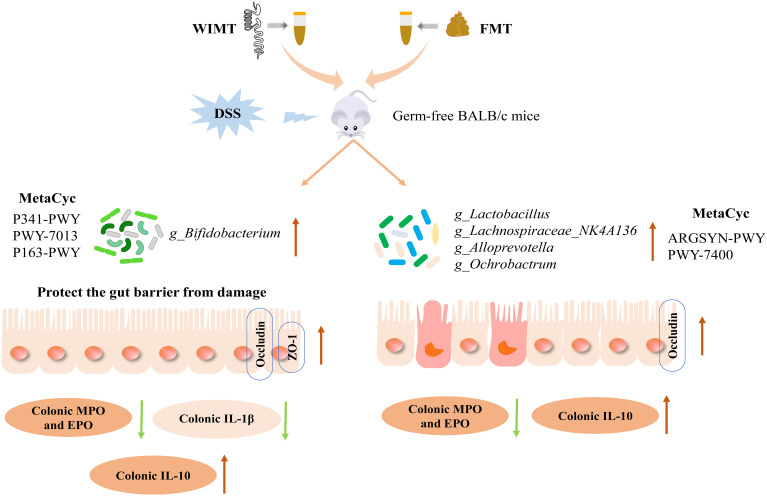

## Introduction

Inflammatory bowel disease (IBD), which comprises Crohn’s disease (CD) and ulcerative colitis (UC) (1), is a chronic, fatal condition that primarily manifests as diarrhea, stomach pain, and rectal bleeding (2, 3). At present, IBD affects up to 0.5% of the population in the West, and this number is projected to continue to rise over the next ten years (2, 4). Additionally, China, a newly industrialized nation with a sizable population, may eventually see more cases of IBD than the West due to its move toward urbanization and westernization (2). Thus, IBD is a global illness (4).

Owing to advancements in next-generation sequencing methods, numerous variations in the gut microbiota’s composition and community have been reported in patients with IBD. The most common finding in IBD is a reduction in bacterial diversity, with a reduced abundance of Firmicutes, and an increased abundance of Proteobacteria, although some of the results linked to dysbiosis in IBD vary among studies due to changes in sample type, survey techniques, patient state, and pharmacological therapy (5–8). Probiotic therapy and other dysbiosis-correcting therapies, such as fecal microbiota transplantation (FMT), have shown promise for treating IBD (9). FMT is a therapeutic approach to ameliorate the abnormal microbial composition of the gut by delivering a patient with fecal microbiota from a healthy donor (9, 10). It has been emphasized as a treatment for correcting dysbiosis in IBD (9). FMT was originally used to treat UC in 1989 (11) and numerous clinical studies have demonstrated that it can dramatically reduce UC symptoms (12, 13). To increase the effectiveness of FMT intervention in UC, it is necessary to further investigate the active ingredients of fecal donors and the mechanism of action of FMT (13, 14). In addition, the composition of donor flora affects the efficacy of FMT in UC, indicating that variations in the composition of donor fecal flora are one of the factors affecting the therapeutic efficacy of FMT (15).

To date, most FMT have relied on stool samples from healthy donors (16). However, different gut segment microbes may also be involved in regulating host health (17). Less commonly, the topic of gut bacteria, both small and large intestinal microbes, has been investigated. By comparing the differences between FMT and whole intestinal microbiota transplantation (WIMT) in reshaping the community structure in germ-free (GF) mice, Li et al. found that WIMT better reproduced the donor microbiota structure and reduced the inflammatory response of the host compared to FMT (18). Given the link between microbiota and IBD, the effect of WIMT intervention in IBD is worth examining. It is possible to explore the connection between IBD and microbiota in GF animals because their microbial backgrounds are well understood (19). Therefore, to explore the differences in the efficacy of WIMT and FMT intervention in colitis mice, we used GF mice colonized with whole intestinal flora and fecal flora followed by dextran sodium sulfate (DSS) to induce the development of colitis in mice. This study provides basic data to support pertinent clinical applications.

## Materials and methods

### Preparation for WIMT and FMT

Fresh feces and contents of whole intestinal segments (all contents of the jejunum, ileum, cecum, and colon) were collected from 8-week-old male SPF BALB/c mice (from the Experimental Animal Center of Huazhong Agricultural University) were collected in sterile fecal collector under anaerobic conditions (80% N_2_, 10% H_2_, 10% CO_2_), and the WIMT and FMT donor samples were homogenized at a ratio of 1:10 (m/v) to sterile saline glycerol buffer (15% glycerol concentration) and mixed well. After the samples were fully dissolved using coarse filtration with sterilized gauze, the bacterial solution was finally obtained by filtration through a 100 μm filter membrane (20).

### Experimental animals and treatments

**Figure 1:**
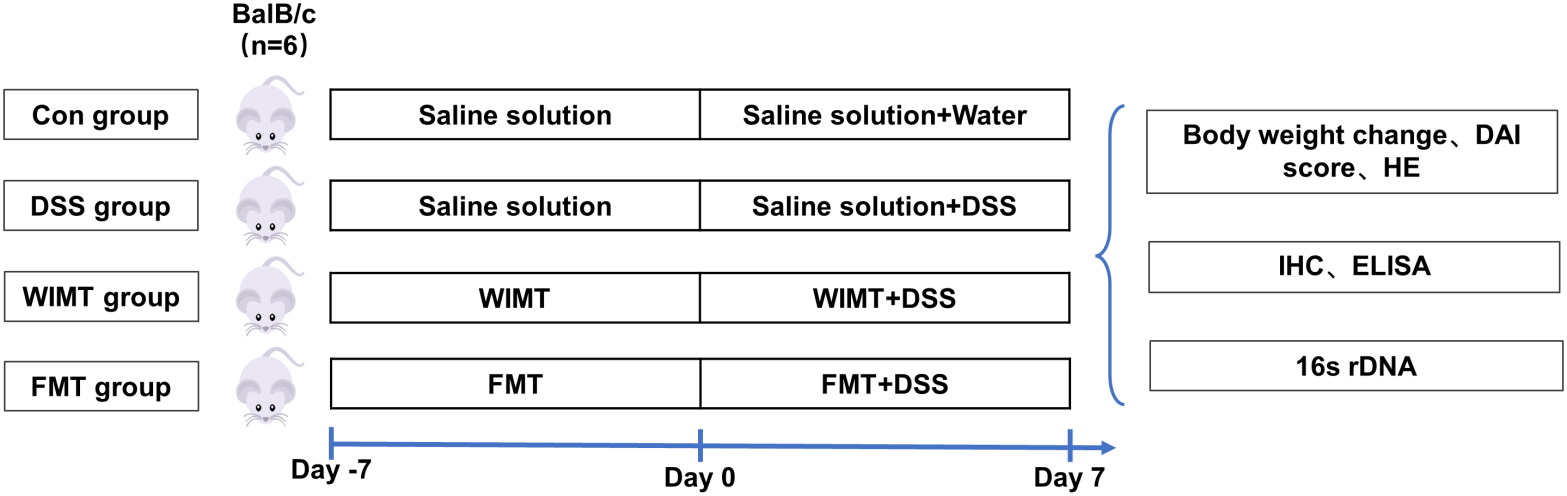
Study design.

Female GF BALB/c mice at 8-10 weeks were obtained from the Germ-Free Animal Platform of Huazhong Agricultural University. Mice were housed in a sterile environment (temperature 25 ± 2°C; relative humidity 45-60%; photoperiod 12h/d; light hours 06:30-18:30), and had free access to sterilized food and water. To study the effects of WIMT and FMT transplantation on the development of UC, the mice were divided into Control, DSS, WIMT, and FMT groups. The mice were instilled with 100 μL of bacterial solution or saline daily; the experimental design is shown in **Figure 1**. Body weight was recorded daily during the experiment to calculate the difference between the weight on the day of measurement and that on day 0 (21). At the end of the experiment, the feces of the mice were collected inside the isolator. The colon tissue, small intestinal contents (jejunal and ileal contents), and large intestinal contents (cecum and colon contents) were collected after the experimental mice were euthanized. Blood was immediately collected from the eyeball, and the separated serum was frozen at -80°C until analysis. All experimental methods were performed according to the Huazhong Agricultural University of Health Guide for the Care and Use of Laboratory Animals. The animal experiment ethics number for this study is HZAUMO-2023-0026.

### Disease activity index

The Disease Activity Index (DAI) includes weight loss score, fecal bleeding score, and stool traits, as shown in **Table 1** (22, 23). Briefly, DAI was measured by weight change (no change = 0; 1-5% = 1; 5-10% = 2; 10-15% = 3; >15% = 4), fecal bleeding score (normal colored stool = 0; brown stool = 1; red stool = 2; bloody stool = 3; heavy bleeding = 4), and fecal traits (normal stool, good shape = 0; soft stool, soft stool adhering to the anus = 1-2; diarrhea, adherent anal = 3-4), which were determined by averaging the three scores (see **Table 1**).

**Table 1:** Disease activity index.

Score	Weight loss (%)	Stool consistency	Bloody stool score
0	None	Normal	Normal colored stool
1	1-5	Loose stool	Brown stool
2	5-10	Loose stool	Reddish stool
3	10-15	Diarrhea	Bloody stool
4	> 15	Diarrhea	Gross bleeding

Disease activity index (DAI), mean score of weight loss, stool consistency, and bloody stool score.

### Mice colon histologic analysis

Distal colon segments from each group of mice were fixed with 4% paraformaldehyde, paraffin-embedded, and cut into 4 μm-thick sections. Sections were stained with hematoxylin-eosin (H&E) and immunohistochemistry (IHC), and images were acquired under a microscope (Nikon Eclipse 80i, Japan). The H&E-stained sections were examined for the degree of inflammatory cell infiltration and tissue damage, and intestinal damage was assessed for the degree of infection, extent of infection, crypt damage, and extent of mucosal involvement (24) (see **Table 2**). The expression of ZO-1 (Proteintech Group, Inc. 21773-1-AP) and occludin (Proteintech Group, Inc. 27260-1-AP) was detected by immunohistochemistry according to the manufacturer’s instructions using Image Pro Plus 6.0 (Media Cybernetics, Inc.) for statistical analysis of mean optical density.

**Table 2:** Histological grading of colitis.

Grade	Inflammation	Extent	Crypt damage	Percent involvement
0	None		None	0
1	Slight	Mucosa	Basal 1/3 damage	1%-33%
2	Moderate	Mucosa and Submucosa	Basal 2/3 damage	34%-66%
3	Severe	Transmural	Entire crypt and epithelium lost	67%-100%

### Enzyme-linked immunosorbent assay

The kits were purchased from Shanghai Enzyme-linked Biotechnology Co. Ltd. (Shanghai, China). All assays were performed according to the manufacturer’s instructions, and IL-1β (ml063132), IL-6 (ml002293), IL-8 (ml001856), IL-17A (ml037864), TNF-α (ml002095), myeloperoxidase (MPO) (ml002070), eosinophil peroxidase (EPO) (ml769125), and serum diamine oxidase (DAO) (ml002070) concentrations were determined by ELISA. Tissue processing for ELISAs are as follows, 1g of tissue sample was weighed and 9ml of PBS (pH 7.2-7.4) was added to homogenise the sample. Centrifuge for about 20 minutes (2000-3000 rpm) and carefully collect the supernatant for testing. Briefly, the kits’ included diluent buffer was used to dilute both standards and samples. A microtitre plate with an antibody precoated in each well was then filled with 100 μL of the sample or standard in duplicate. Diluent buffer was used as a negative control. The plates were incubated for 2 h at 37°C. After incubation, 100 μL of biotin antibody was added to each well after removing the liquid and incubated for 1 h at 37°C. The wells were washed 3 times with 200 μL volume of wash buffer. Next, each well received 100 μL of horseradish peroxidase-avidin for 1 hour at 37°C. After a final wash, 90 μL of the supplied TMB substrate was added and incubated for 30 min in the dark at 37°C. 50 μL of the supplied stop solution was used to stop the reaction. Absorbance was measured at 450 nm using a plate reader (BioTek Instruments, Inc.), and the levels of cytokines in the samples was calculated from the standard curve.

### D-lactate measurements

The levels of DAO and D-lactic acid (D-LA) in serum were tested for permeability. D-LA (ml158174) was determined by spectrophotometry using a D-Lactic acid detection kit (Shanghai Enzyme-linked Biotechnology Co. Ltd.). Briefly, the visible spectrophotometer (Shanghai Enzyme-linked Biotechnology Co. Ltd.) is preheated for more than 30min, the wavelength is adjusted to 450nm and zeroed with distilled water. The reagents and samples were added sequentially to 1mL glass cuvette according to the reagent manufacturer’s instructions, mixed and immediately reacted in the dark at 37°C for 30min, and the absorbance value was read at 450nm. The levels of D-LA in the sample were calculated from the standard curve.

### 16S rDNA sequencing and analysis

Samples were collected inside the isolator, immediately transported on ice to a −80°C refrigerator, and transported on dry ice for amplicon sequencing. Genomic DNA was extracted by the Cetyltrimethylammonium Bromide (CTAB) method and then amplified using specific primers with barcode (515F-806R for 16S V4 region) after assessing the purity and concentration of DNA. The PCR products were mixed in equal concentrations according to the concentration of the PCR product and then purified by electrophoresis using 1×TAE on a 2% agarose gel. Sequences with a primary band size between 400-450 bp were selected and the gel was cut to recover the target bands. Libraries were constructed using an Illumina TruSeq DNA PCR-Free Library Preparation Kit. The libraries were sequenced using NovaSeq 6000 after they were qualified using Qubit quantification and library testing. The analysis was carried out by splitting each sample from the downstream data based on barcode sequences and PCR amplification primer sequences, and the reads were spliced using FLASH software (V1.2.11, http://ccb.jhu.edu/software/FLASH/) after truncating the barcode and primer sequences to obtain Raw Tags. Raw Tags were then quality-controlled using the Fastp software to obtain high-quality Clean Tags. Finally, Clean Tags were compared to the database using Usearch software to detect chimeras and remove them (25) to obtain the final Effective Tags, and the DADA2 module or deblur in QIIME2 software (26) (https://qiime2.org/) was used for a module or deblur for noise reduction (27) and filtering out low-quality sequences to obtain the final Amplicon Sequence Variants (ASVs) and feature-table. The ASVs were then compared to the Silva database (V138-99) using the classify-sklearn module in R software to obtain species information for each ASV. Alpha diversity: R was used to calculate Shannon, Simpson, and Pielou indices and analyze the inter-group differences in alpha diversity. Beta diversity: Jaccard distances were calculated using R. The PCoA was plotted using the vegan package (versions 2.6-2) in R. Subsequently, the adonis function in R was used to analyze the significance of differences in community structure between groups. Functional annotation: Prediction of colony metabolic function using PICRUSt2. Distinctive and shared features and linear discriminant analysis effect size (LEfSe, p < 0.05, LDA > 2.0) were completed using Wekemo Bioincloud (https://www.bioincloud.tech).

### Statistical methods

Data were analyzed using GraphPad Prism 6 (GraphPad Software, LLC), and the Student’s t-test was used for statistical analysis between the two groups. A non-parametric test was used to analyse count data with Kruskal-Wallis test followed by Dunn’s multiple comparisons test. One-way ANOVA and Bonferroni’s multiple comparison test were performed on data from more than two groups, and the data are expressed as mean ± SEM. The differences were considered statistically significant at p < 0.05.

## Results

### WIMT is more effective in relieving DSS-induced acute colitis

**Figure 2:**
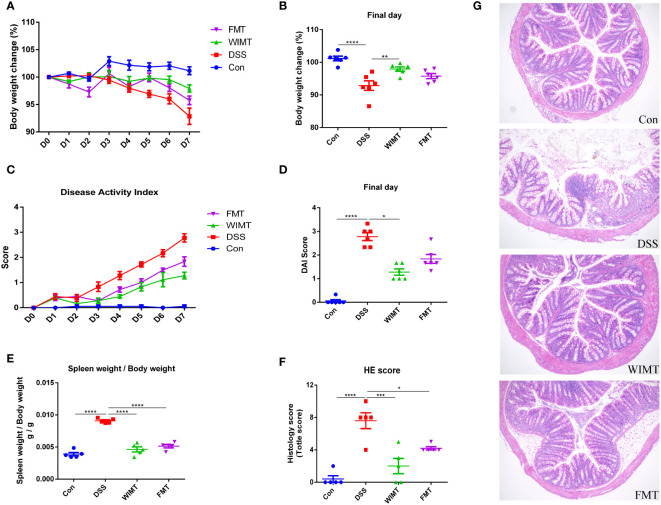
WIMT and FMT alleviated DSS-induced colitis to different extents. **(A)** Body weight change; **(B)** Body weight change on the final day; **(C)** DAI score; **(D)** DAI score on the final day; **(E)** Ratio of spleen weight to body weight; **(F)** Histological score; **(G)** H&E staining of colon tissue (100×). * p ≤ 0.05, ** p ≤ 0.01, *** p ≤ 0.001, **** p ≤ 0.0001, data are represented as mean ± SEM.

To investigate the effects of WIMT and FMT on colitis, mice with 3% DSS-induced colitis were treated with WIMT, FMT, and saline. The body weight of mice in the DSS group decreased significantly, and those of mice in the WIMT group decreased less than those of the mice in the FMT group. On the final day of the experiment, the body weights of mice in the FMT group were not statistically different compared with the DSS group, while the mice in the WIMT group were significantly heavier than those in the DSS group (p = 0.0056) (**Figures 2A, B**). WIMT and FMT intervention reduced DSS-induced DAI scores, with no significant difference on the last day DAI between the WIMT and FMT groups (**Figures 2C, D**). Mice in both groups with microbiota transplantation had lower weight of the spleen weight to body weight (**Figure 2E**). Histological staining showed that DSS treatment resulted in different degrees of histological damage in each group of mice (WIMT vs. DSS, p = 0.0002; FMT vs. DSS, p = 0.0163). Compared with the DSS group, donor fluids in both groups showed reduced inflammatory cell infiltration and mucosal damage in colonic tissue and reduced histological scores, with the lowest scores in the WIMT group, however, no significant difference was observed between the WIMT and FMT groups (**Figures 2F, G**). In summary, these results suggest that pre-colonization of both donor flora could alleviate DSS-induced colitis, with WIMT having a higher intervention efficacy than FMT.

### Effect of WIMT and FMT on colonic inflammatory markers and cytokines

**Figure 3:**
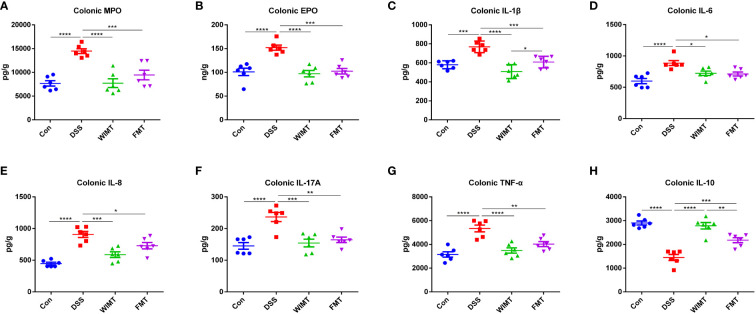
Effect of WIMT and FMT on the inflammatory response in DSS-induced colitis. **(A)** Colonic MPO; **(B)** Colonic EPO;**(C)** Colonic IL-1β; **(D)** Colonic IL-6; **(E)** Colonic IL-8; **(F)** Colonic TNF-α; **(G)** Colonic IL-17A; **(H)** Colonic IL-10. * p ≤ 0.05, ** p ≤ 0.01, *** p ≤ 0.001, **** p ≤ 0.0001, data are represented as mean ± SEM.

To explore the effects of the two donors flora on immune homeostasis and inflammatory markers of colitis, the levels of pro-inflammatory factors IL-1β, IL-6, IL-8, IL-17A, TNF-α, anti-inflammatory factor IL-10, and colitis markers MPO and EPO in colonic tissues were measured by ELISA. As shown in **Figures 3A, B**, WIMT and FMT interventions significantly reduced the levels of MPO (WIMT vs. DSS, p < 0.0001; FMT vs. DSS, p = 0.0008) and EPO (WIMT vs. DSS, p < 0.0001; FMT vs. DSS, p = 0.0001) in colonic tissues compared to those in the DSS group, and there was no significant difference between the WIMT and FMT groups. WIMT and FMT interventions significantly reduced the levels of pro-inflammatory factors IL-1β (WIMT vs. DSS, p < 0.0001; FMT vs. DSS, p = 0.0007), IL-6 (WIMT vs. DSS, p = 0.022; FMT vs. DSS, p = 0.0133), IL-8 (WIMT vs. DSS, p = 0.0002; FMT vs. DSS, p = 0.034), IL-17A (WIMT vs. DSS, p = 0.0003; FMT vs. DSS, p = 0.0011), and TNF-α (WIMT vs. DSS, p < 0.0001; FMT vs. DSS, p = 0.0034) in colonic tissues (**Figures 3C-G**) and increased IL-10 levels (WIMT vs. DSS, p < 0.0001; FMT vs. DSS, p = 0.0009) (**Figure 3H**). IL-1β levels were significantly lower in the WIMT group compared to the FMT group (p = 0.0345), while IL-10 levels were higher in the WIMT group (p = 0.0052). In summary, WIMT intervention alleviated the symptoms of colitis in mice by improving cytokine homeostasis.

### Interventions using WIMT and FMT prevent the deterioration of gut barrier function brought on by DSS

**Figure 4:**
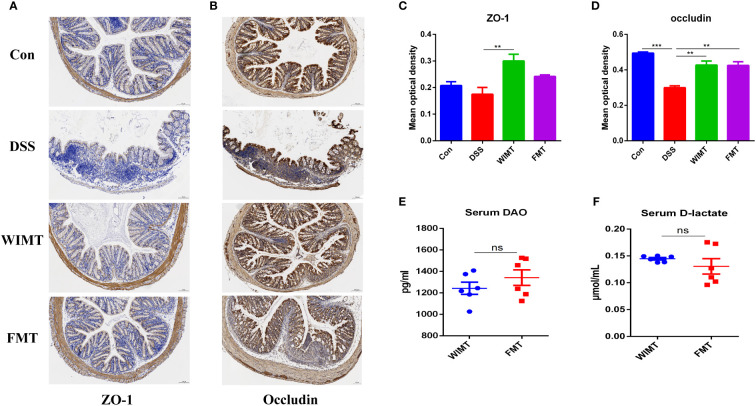
Effect of WIMT and FMT on the intestinal barrier in DSS-induced colitis. Immunohistochemistry for ZO-1 **(A)** and occludin **(B)** in each group (100μm, n = 3); **(C, D)** Average optical density. The changes of levels of serum DAO **(E)** and D-lactate **(F)**. ** p ≤ 0.01, *** p ≤ 0.001, data are represented as mean ± SEM. ns, no significance difference.

Since dysfunction of the intestinal epithelial barrier is associated with IBD, we hypothesized that both donor group would alleviate DSS-induced damage by protecting the intestinal barrier. We analyzed the average optical density (AOD) of tight junction proteins ZO-1 and occludin in four groups of mouse colonic tissues using IHC. As shown in **Figures 4A-D**, the AOD of colonic ZO-1 was significantly higher in the WIMT group than in the DSS group (p = 0.008), while there was no significant difference between the FMT and DSS groups. In addition, the AOD of occludin was significantly higher in both the WIMT and FMT groups than that in the DSS group (WIMT vs. DSS, p = 0.0029; FMT vs. DSS, p = 0.0031). To investigate the barrier mechanism underlying the difference in anti-colitis efficacy between the WIMT and FMT groups, we also evaluated serum DAO and D-LA levels linked with intestinal permeability, and there was no statistically significant difference between the two groups (**Figures 4E, F**). In summary, both donor group alleviated DSS-induced damage by maintaining intestinal barrier integrity.

### Analysis of microbial diversity in the WIMT and FMT groups

**Figure 5:**
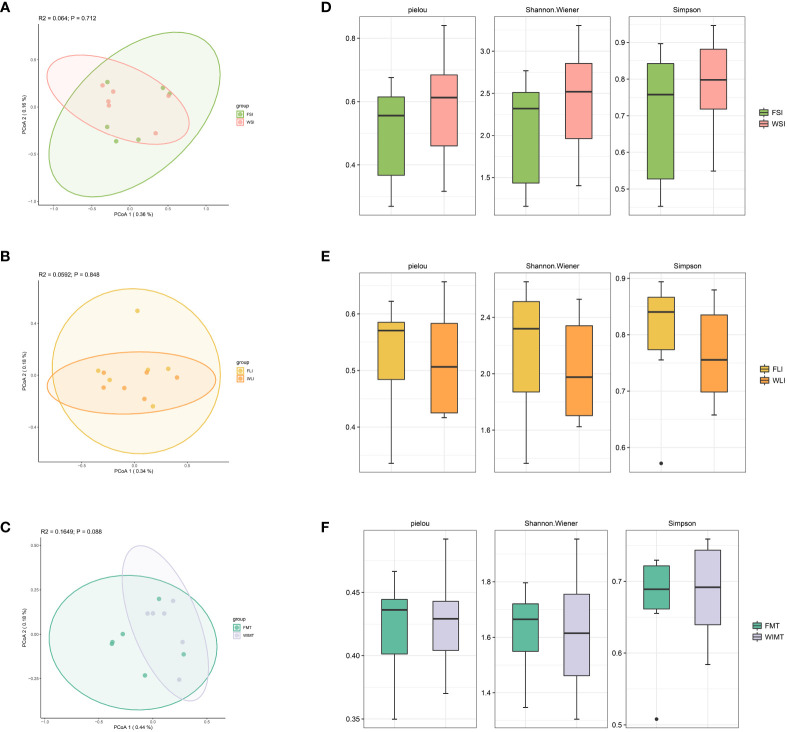
Effect of WIMT and FMT on the microbiota diversity in DSS-induced colitis. **(A)** PCoA analysis for FSI vs WSI groups; **(B)** PCoA analysis for FLI vs WLI groups;**(C)** PCoA analysis for FMT vs WMIT groups; **(D)** Alpha diversity of FSI and WSI groups; **(E)** Alpha diversity of FLI and WLI groups;**(F)** Alpha diversity of FMT and WIMT groups.

Considering the relationship between microbiota and IBD, we analyzed the microbial diversity in the small intestine (jejunal and ileal contents), large intestine (cecum and colon contents), and feces in the WIMT and FMT groups using 16S rDNA. To assess the beta diversity of the community, we employed the Jaccard index. There was no significant difference between the small intestinal community structure of the WIMT (WSI) and the FMT groups (FSI) (**Figure 5A**). The large intestinal community structure of the WIMT group (WLI) was not significantly different from that of the FMT group (FLI) (**Figure 5B**). Furthermore, the fecal community structure of the WIMT group was not significantly different from that of the FMT group (**Figure 5C**). The Pielou, Shannon indices, and Simpson were used to evaluate alpha diversity (**Figures 5D-F**), and there was no significant difference between the two groups. These findings showed that differences in efficacy may not be related to the indices.

### Common and different microorganisms between the WIMT and FMT groups

**Figure 6:**
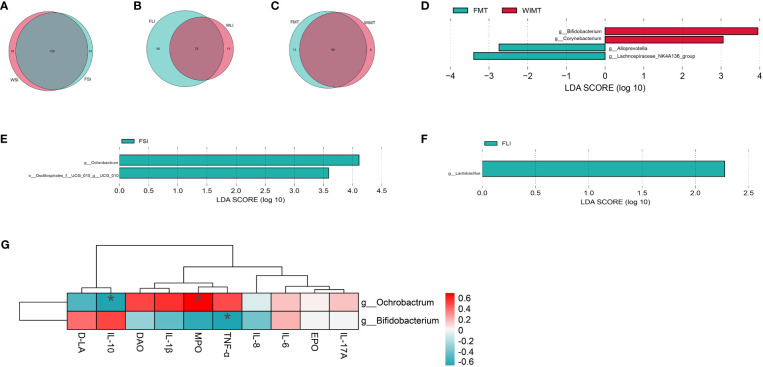
Differences in microbes of the WIMT and FMT groups. **(A)** Venn diagram for FSI and WSI groups; **(B)** Venn diagram for FLI and WLI groups;**(C)** Venn diagram for FMT and WMIT groups; **(D)** LEfSe analysis of WIMT and FMT groups; **(E)** LEfSe analysis of FSI and WSI groups; **(F)** LEfSe analysis of FLI and WLI groups; **(G)** Correlation analysis of differential bacteria with cytokines. * p ≤ 0.05.

By examining the shared genera, we were able to explain the anti-inflammatory effects in both groups. A Venn diagram was plotted showing a total of 129 genera of small intestinal flora (**Figure 6A**, **Supplement Table 1**), 76 genera of large intestinal flora (**Figure 6B**, **Supplement Table 2**), and 60 genera of fecal flora (**Figure 6C**, **Supplement Table 3**) in the two groups of mice. The differences in microorganisms between the two groups were analyzed using LEfSe (LDA > 2) to explain the superior efficacy of the WIMT group. The results showed that *Lachnospiraceae_NK4A136_group* and *Alloprevotella* (enriched in feces, **Figure 6D**), *Ochrobactrum* (enriched in the small intestine, **Figure 6E**), and *Lactobacillus* (enriched in the large intestine, **Figure 6F**) were significantly enriched in the FMT group, while *Bifidobacterium* and *Corynebacterium* (enriched in feces, **Figure 6D**) were significantly enriched in the WIMT group. The correlation between various bacteria and cytokines is displayed on a heat map (**Figure 6G**). Bacteria *Bifidobacterium* enriched in the WIMT group was negatively correlated with pro-inflammatory factors TNF-α (p = 0.04264557), and Bacteria *Ochrobactrum* enriched in the FMT group was positively correlated with inflammatory biomarkers MPO (p = 0.011331713) and negatively correlated with anti-inflammatory factors and IL-10 (p = 0.041346). In conclusion, there were significant differences in the microbiota between the WIMT and FMT groups, and these differences were associated with the effectiveness of the intervention.

### Functional differences in the WIMT and FMT groups

**Figure 7:**
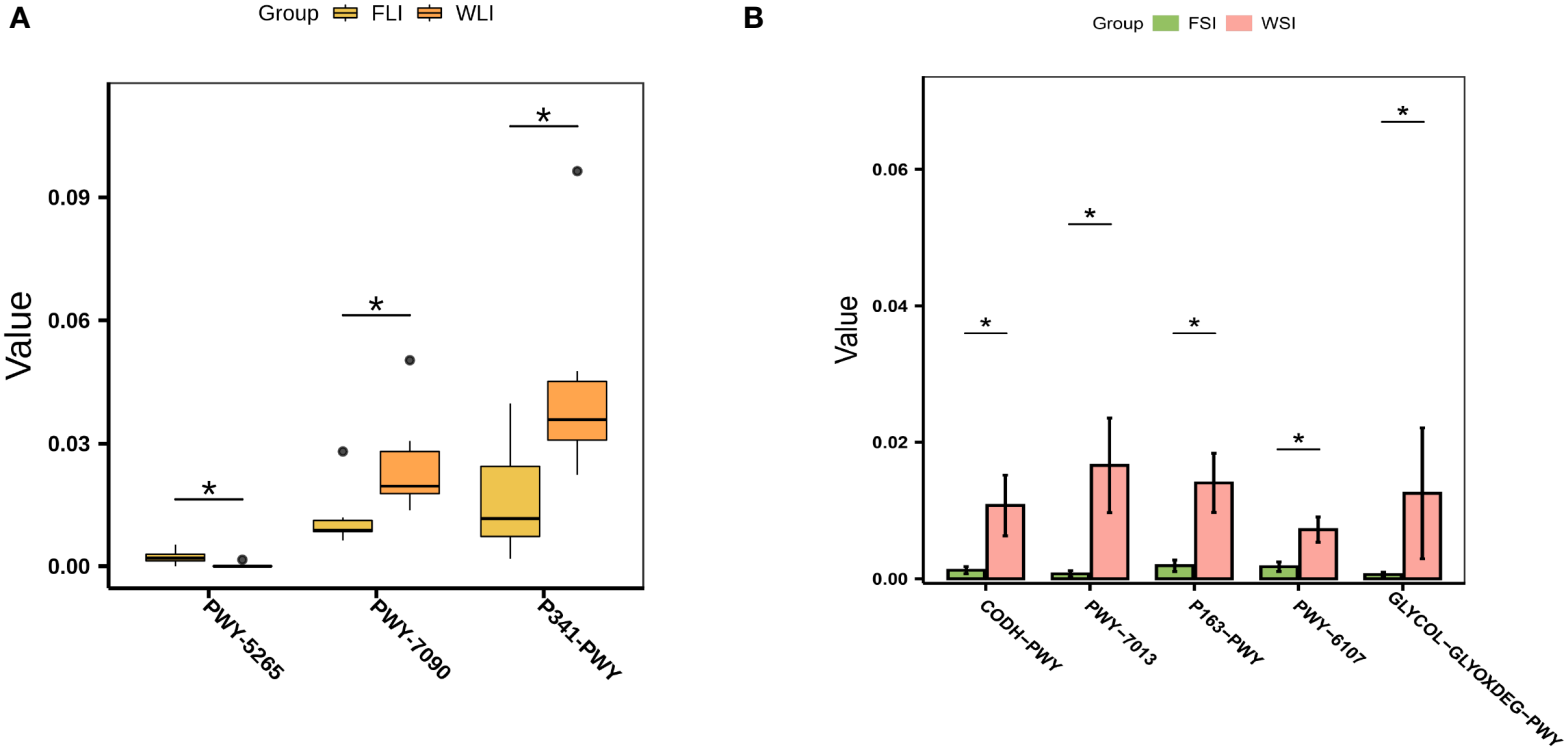
Effect of WIMT and FMT on the microbial functional in DSS-induced colitis. **(A)** Differential metabolic pathways between the FLI and WLI groups; **(B)** Differential metabolic pathways between FSI and WSI groups. * p ≤ 0.05.

To further clarify the mechanism of superior efficacy in the WIMT group at the functional level, we used PICRUSt2 to predict variations in microbially engaged metabolic pathways (MetaCyc database) in the small intestine, large intestine, and feces between the WIMT and FMT groups. The metabolic pathways, such as peptidoglycan biosynthesis II (staphylococci) (PWY-5265), L-arginine biosynthesis I (ARGSYN-PWY), and L-arginine biosynthesis IV (PWY-7400), were significantly enriched in the FMT group (**Figures 7A, B**, **Supplement Table 4**). In contrast, metabolic pathways, such as L-lysine fermentation to acetate and butanoate (P163-PWY), glycolysis V (P341-PWY), propylene glycol degradation (PWY-7013), and L-histidine degradation I (HISDEG-PWY), were significantly enriched in the WIMT group (**Figures 7A, B**, **Supplement Table 4**). The results indicated that the differences in efficacy between the WIMT and FMT groups were related to different metabolic pathways.

## Discussion

IBD is a widespread condition that deteriorates the quality of life of many people (4). Several studies have reported alterations in the community and structure of the gut microbiota in patients (5–8). FMT has been utilized as an innovative and successful therapy to treat IBD by restoring the diversity and composition of gut microbiota (9). Previous research has shown that WIMT, as opposed to FMT, can more accurately reproduce the community structure and suppress the host’s inflammatory response (18). However, it is not clear whether WIMT is more effective in the intervention of IBD. In this study, the effects of WIMT and FMT on the susceptibility of DSS-induced colitis were examined. The trial findings demonstrated that by preventing weight loss and lowering the DAI and histological scores, both WIMT and FMT reduced the symptoms of colitis. Both groups of donors showed downregulated levels of inflammatory biomarkers MPO (28, 29) and EPO (30) and regulated homeostasis of colonic inflammatory factors. It was demonstrated that both groups of GF mice had decreased susceptibility to DSS and that the WIMT intervention efficacy was superior to that of the FMT group.

As with many other tissue injuries, IBD is characterized by distorted expression of inflammatory cytokines. Intestinal inflammation is regulated by cytokines IL-1β, IL-6, IL-8, TNF-α, and IL-10 (31–33). Increased levels of TNF-α, IL-1β, and IL-6 have been linked to intestinal dysfunction (34). By recruiting granulocytes and activating CD4^+^ T cells in IBD, IL-1β stimulates the production of pro-inflammatory molecules, such as IL-17A, IL-12, and IFN-γ, aggravating intestinal inflammation (35, 36). Secretion of the anti-inflammatory factor IL-10 ameliorates mucosal damage in IBD and protects lymphocytes, which inhibits IBD by suppressing the host autoimmune response (37, 38). In the present study, both WIMT and FMT significantly inhibited the expression of DSS-induced pro-inflammatory factors. The levels of IL-1β were significantly lower in the WIMT group than in the FMT group, and the levels of IL-10 were significantly higher in the WIMT group than in the FMT group, indicating that the WIMT group had better control over cytokine homeostasis. Intestinal epithelial barrier dysfunction is associated with IBD (39), and the epithelial cytoskeleton, which is composed of tight junction proteins such as ZO-1 and occludin, plays an important role in maintaining intestinal mucosal barrier function and regulating intestinal permeability (40–42). D-LA and DAO levels, which represent intestinal permeability, are significantly higher in patients with IBD (43). In this study, WIMT intervention significantly elevated ZO-1 and occludin expression levels in colonic tissues, and FMT intervention significantly elevated occludin expression levels in colonic tissues, and there was no significant difference in intestinal permeability between the WIMT and FMT groups. In conclusion, both WIMT and FMT could protect the intestinal barrier by regulating inflammatory factor homeostasis to lessen the damage caused by DSS.

It has been demonstrated that intestinal flora is essential for the development or remission of IBD (44). The microbiota sequencing data from this investigation revealed the presence of numerous shared genera in both groups of mice, such as *Eubacterium_hallii_group* in the small intestinal tract, *Bifidobacterium* in the large intestinal tract, and *Parabacteroides* in the feces. It has been reported that genera *Eubacterium_hallii_group*, *Bifidobacterium*, and *Parabacteroides* possess anti-inflammatory efficacy (45–47), and in the present study, these shared genera were associated with anti-inflammatory efficacy in both groups. *Bifidobacterium*, which improves the intestinal community structure and resists gastrointestinal inflammation (45), was considerably enriched in the WIMT group. The expression of tight junction proteins and the mucin family was upregulated by *Bifidobacterium adolescentis*, which belongs to the genus *Bifidobacterium* and regulates intestinal inflammation by reducing pro-inflammatory cytokines such as IL-6 and IL-1β, increasing the level of IL-10, and increasing Treg and Th2 cells in the lamina propria of the colon to inhibit the overgrowth of harmful bacteria (44). *Bifidobacterium bifidum* targets the Toll-like receptor 2 pathway in an NF-*κ*B non-dependent manner, which enhances the intestinal epithelial tight junction barrier and prevents intestinal inflammation (48). Clinical research and animal experiments have demonstrated that *Bifidobacterium longum* can reduce the signs of chronic inflammation and colitis (49, 50). In the current study, the enrichment of *Bifidobacterium* in the WIMT group was significantly negatively correlated with the pro-inflammatory factor TNF-α, and the WIMT group in this study had superior efficacy compared to the FMT group as a result of *Bifidobacterium* enrichment.

*Ochrobactrum*, *Lactobacillu*s, *Alloprevotella*, and *Lachnospiraceae NK4A136_group* were significantly enriched in the FMT group. *Lactobacillus* is a potential catalyst for the development of the human immune system (51). Together, prebiotics and *Lactobacillus* dramatically reduced the symptoms of UC in clinical investigations (52). Members of the genus *Lactobacillus* also function as probiotics. By controlling oxidative stress and immunological responses, *Lactobacillus plantarum* effectively protects against DSS-induced IBD in mice (53). Widespread in the intestines of healthy individuals, *Lactobacillus reuteri* regulates the intestinal immune system and can reduce inflammation *via* a number of processes (54). Butyrate can be produced by members of *Lachnospiraceae* (55), and it is not only a major source of energy for intestinal epithelial cells but also inhibits pro-inflammatory cytokine signaling pathways (56). *Lactobacillus* and *Lachnospiraceae NK4A136*, which were enriched in the FMT group, contributed to the anti-inflammatory efficacy of FMT. The abundance of *Alloprevotella* was found to be higher in the AOM/DSS-treated group than in the control group in a study by Wang et al. (57), raising the possibility that it may be related to the severity of colitis. According to a study by Walujkar et al., *Ochrobactrum* was substantially more abundant in the microbiota during the aggravated phase of UC than it in the remission phase, which helps identify the particular genera that dominate the microbiota during the disease (58). In the current study, the enriched *Ochrobactrum* in the FMT group was significantly positively correlated with the inflammatory marker MPO and significantly negatively correlated with the anti-inflammatory factor IL-10, which led to its poorer efficacy than that of WIMT.

Short-chain fatty acids (SCFAs; mainly acetate, propionate, and butyrate) produced by intestinal bacteria can modulate protective immunity and reduce tissue inflammation (59, 60). In this study, functional prediction analysis based on the MetaCyc database using PICRUSt2 revealed that glycolysis V (Pyrococcus, P341-PWY), propylene glycol degradation (PWY-7013), and lysine degradation to acetate and propionate (P163-PWY) pathways were significantly enriched in the WIMT group. The glycolytic V pathway produces pyruvate, which can be further converted to acetate in this metabolic pathway (61–63). Propylene glycol can be degraded by bacteria to produce propionate (64). Bacteria involved in lysine metabolism produce acetate and propionate in L-lysine fermentation through the acetate and butanoate pathways (65), and all three metabolic pathways enriched in the WIMT group may be involved in the production of SCFAs, which is related to its anti-inflammatory efficacy. Amino acids play a role in the prevention and treatment of IBD by regulating the physiological activity of intestinal epithelial cells and by protecting the intestinal barrier (66). The addition of arginine prevents LPS-induced oxidative damage and apoptosis (67). In this study, L-arginine biosynthesis I (ARGSYN-PWY) and L-arginine biosynthesis IV (PWY-7400) were significantly enriched in the FMT group, which could be associated with the FMT group’s ability to prevent inflammation and preserve the intestinal barrier.

There are some limitations of our study. Both WIMT and FMT were pre-colonized into mice and intervened continuously, thus reducing susceptibility to colitis. Further studies are needed to explore the differences in therapeutic efficacy between the two groups. In addition, the causal relationship between specific bacteria and IBD in animals or in *in vitro* experiments and the underlying molecular mechanisms need to be further investigated.

In conclusion, both WIMT and FMT could significantly reduce the susceptibility of mice to DSS-induced colitis by providing probiotics, protecting the intestinal mucosal barrier, and regulating the dynamic balance of cytokines. Notably, the mice in the WIMT group responded better to the intervention than those in the FMT group, which may be attributable to the enrichment of metabolic pathways involving SCFAs and *Bifidobacterium* in the WIMT group. This study provides new insights into microecological interventions for IBD.

## Data availability statement

The raw data supporting the conclusions of this article will be made available by the authors, without undue reservation. The datasets presented in this study can be found in online repositories. Data is deposited in China National Microbiology Data Center (NMDC) with accession number NMDC10018297 (https://nmdc.cn/).

## Ethics statement

The animal study was reviewed and approved by Institutional Animal Care and Use Committee of the Huazhong Agricultural University.

## Author contributions

YY, ST, and HW designed the experiment. YY, JH, YW, LL, ZZ, XT, HZ, ZW, MD, JZ, SF, and WC performed the animal trials, sample collection, and data analysis. YY drafted the manuscript. ST, QC, and HW revised the manuscript. All authors contributed to the article and approved the submitted version.
