# Supplementary material for: Whole intestinal microbiota transplantation is more effective than fecal microbiota transplantation in reducing the susceptibility of DSS-induced germ-free mice colitis
Source: Front Immunol. 2023 May 9;14:1143526. doi: 10.3389/fimmu.2023.1143526 (PMC10206398; doi:10.3389/fimmu.2023.1143526)
Supplement: Supplementary file 1 [file Table_1.docx]

**Supplement Table1.** WSI and WLI common features

| **Features** | **SUM_Abundance** |
| --- | --- |
| d__Bacteria;p__Firmicutes;c__Clostridia;o__Lachnospirales;f__Lachnospiraceae;g__Lachnospiraceae_FCS020_group | 0.013846 |
| d__Bacteria;p__Firmicutes;c__Clostridia;o__Lachnospirales;f__Lachnospiraceae;__ | 1.200607 |
| d__Bacteria;p__Firmicutes;c__Clostridia;o__Lachnospirales;f__Lachnospiraceae;g__Lachnospiraceae_NK4A136_group | 0.349113 |
| d__Bacteria;p__Actinobacteriota;c__Coriobacteriia;o__Coriobacteriales;f__Eggerthellaceae;g__Enterorhabdus | 0.015028 |
| d__Bacteria;p__Firmicutes;c__Clostridia;o__Oscillospirales;f__Ruminococcaceae;g__Incertae_Sedis | 0.054215 |
| d__Bacteria;p__Bacteroidota;c__Bacteroidia;o__Bacteroidales;f__Muribaculaceae;g__Muribaculaceae | 2.992421 |
| d__Bacteria;p__Firmicutes;c__Bacilli;__;__;__ | 0.000958 |
| d__Bacteria;p__Bacteroidota;c__Bacteroidia;o__Bacteroidales;f__Rikenellaceae;g__Alistipes | 0.813962 |
| d__Bacteria;p__Proteobacteria;c__Alphaproteobacteria;o__Sphingomonadales;f__Sphingomonadaceae;g__Sphingomonas | 0.198879 |
| d__Bacteria;p__Firmicutes;c__Clostridia;o__Clostridia_vadinBB60_group;f__Clostridia_vadinBB60_group;g__Clostridia_vadinBB60_group | 0.038795 |
| d__Bacteria;p__Firmicutes;c__Clostridia;o__Oscillospirales;f__Oscillospiraceae;g__uncultured | 0.06392 |
| d__Bacteria;p__Proteobacteria;c__Alphaproteobacteria;o__Rhodospirillales;f__uncultured;g__uncultured | 0.042572 |
| d__Bacteria;p__Firmicutes;c__Clostridia;o__Lachnospirales;f__Lachnospiraceae;g__GCA-900066575 | 0.019527 |
| d__Bacteria;p__Firmicutes;c__Bacilli;o__Erysipelotrichales;f__Erysipelotrichaceae;g__uncultured | 0.001751 |
| d__Bacteria;p__Firmicutes;c__Clostridia;o__Oscillospirales;f__Oscillospiraceae;g__Colidextribacter | 0.02525 |
| d__Bacteria;p__Chloroflexi;c__Ktedonobacteria;o__C0119;f__C0119;g__C0119 | 0.000302 |
| d__Bacteria;p__Actinobacteriota;c__Actinobacteria;o__Corynebacteriales;f__Corynebacteriaceae;g__Corynebacterium | 0.007775 |
| d__Bacteria;p__Firmicutes;c__Clostridia;o__Lachnospirales;f__Lachnospiraceae;g__Lachnoclostridium | 0.152041 |
| d__Bacteria;p__Firmicutes;c__Clostridia;o__Lachnospirales;f__Lachnospiraceae;g__Blautia | 0.044743 |
| d__Bacteria;p__Firmicutes;c__Bacilli;o__Erysipelotrichales;f__Erysipelotrichaceae;g__Erysipelotrichaceae | 0.01773 |
| d__Bacteria;p__Firmicutes;c__Clostridia;o__Clostridia_UCG-014;f__Clostridia_UCG-014;g__Clostridia_UCG-014 | 0.596515 |
| d__Bacteria;p__Firmicutes;c__Clostridia;o__Oscillospirales;f__Oscillospiraceae;__ | 0.25542 |
| d__Bacteria;p__Firmicutes;c__Clostridia;o__Christensenellales;f__Christensenellaceae;g__uncultured | 0.001048 |
| d__Bacteria;p__Firmicutes;c__Clostridia;o__Clostridiales;f__Clostridiaceae;g__Candidatus_Arthromitus | 0.023434 |
| d__Bacteria;p__Patescibacteria;c__Saccharimonadia;o__Saccharimonadales;f__Saccharimonadaceae;g__Candidatus_Saccharimonas | 0.349514 |
| d__Bacteria;p__Actinobacteriota;c__Actinobacteria;o__Streptomycetales;f__Streptomycetaceae;g__Streptomyces | 0.019321 |
| d__Bacteria;p__Bacteroidota;c__Bacteroidia;o__Bacteroidales;f__Bacteroidaceae;g__Bacteroides | 3.216377 |
| d__Bacteria;p__Firmicutes;c__Clostridia;o__Lachnospirales;f__Lachnospiraceae;g__uncultured | 0.00419 |
| d__Bacteria;p__Firmicutes;c__Clostridia;o__Oscillospirales;f__Oscillospiraceae;g__Oscillibacter | 0.009678 |
| d__Bacteria;p__Bacteroidota;c__Bacteroidia;o__Bacteroidales;f__Marinifilaceae;g__Odoribacter | 0.014581 |
| d__Bacteria;p__Firmicutes;c__Clostridia;o__Lachnospirales;f__Lachnospiraceae;g__Lachnospiraceae_UCG-006 | 0.23237 |
| d__Bacteria;p__Desulfobacterota;c__Desulfovibrionia;o__Desulfovibrionales;f__Desulfovibrionaceae;g__uncultured | 0.049634 |
| d__Bacteria;p__Firmicutes;c__Bacilli;o__Lactobacillales;f__Lactobacillaceae;g__Lactobacillus | 0.001299 |
| d__Bacteria;p__Firmicutes;c__Clostridia;o__Oscillospirales;f__Oscillospiraceae;g__NK4A214_group | 0.004639 |
| d__Bacteria;p__Bacteroidota;c__Bacteroidia;o__Chitinophagales;f__Chitinophagaceae;g__Flavisolibacter | 0.003746 |
| d__Bacteria;p__Firmicutes;c__Clostridia;o__Lachnospirales;f__Lachnospiraceae;g__Acetatifactor | 0.004919 |
| d__Bacteria;p__Firmicutes;c__Clostridia;o__Oscillospirales;f___Eubacterium__coprostanoligenes_group;g___Eubacterium__coprostanoligenes_group | 0.006653 |
| d__Bacteria;p__Firmicutes;c__Clostridia;o__Lachnospirales;f__Lachnospiraceae;g__Marvinbryantia | 0.010172 |
| d__Bacteria;p__Acidobacteriota;c__Acidobacteriae;o__Bryobacterales;f__Bryobacteraceae;g__Bryobacter | 0.001078 |
| d__Bacteria;p__Chloroflexi;c__KD4-96;o__KD4-96;f__KD4-96;g__KD4-96 | 0.000555 |
| d__Bacteria;p__Firmicutes;c__Bacilli;o__Lactobacillales;__;__ | 0.000109 |
| d__Bacteria;p__Firmicutes;c__Clostridia;o__Lachnospirales;f__Lachnospiraceae;g__ASF356 | 0.002002 |
| d__Bacteria;p__Proteobacteria;c__Alphaproteobacteria;o__Rhizobiales;f__Rhizobiaceae;g__Ochrobactrum | 0.001233 |
| d__Bacteria;p__Bacteroidota;c__Bacteroidia;o__Bacteroidales;f__Tannerellaceae;g__Parabacteroides | 0.035677 |
| d__Bacteria;p__Firmicutes;c__Bacilli;o__Bacillales;f__Bacillaceae;g__Bacillus | 0.000378 |
| d__Bacteria;p__Bacteroidota;c__Bacteroidia;o__Bacteroidales;f__Prevotellaceae;g__Prevotellaceae_UCG-001 | 0.001898 |
| d__Bacteria;p__Bacteroidota;c__Bacteroidia;o__Chitinophagales;f__Chitinophagaceae;g__Chitinophagaceae | 0.000609 |
| d__Bacteria;p__Firmicutes;c__Clostridia;o__Oscillospirales;f__Butyricicoccaceae;g__Butyricicoccus | 0.018332 |
| d__Bacteria;p__Actinobacteriota;c__Actinobacteria;o__Corynebacteriales;f__Nocardiaceae;g__Nocardia | 0.001387 |
| d__Bacteria;p__Gemmatimonadota;c__Gemmatimonadetes;o__Gemmatimonadales;f__Gemmatimonadaceae;g__Gemmatimonas | 0.000454 |
| d__Bacteria;p__Actinobacteriota;c__Actinobacteria;o__Bifidobacteriales;f__Bifidobacteriaceae;g__Bifidobacterium | 0.059509 |
| d__Bacteria;p__Firmicutes;c__Bacilli;o__Erysipelotrichales;f__Erysipelatoclostridiaceae;g__Erysipelatoclostridium | 0.018141 |
| d__Bacteria;p__Firmicutes;c__Clostridia;o__Peptococcales;f__Peptococcaceae;g__uncultured | 0.001134 |
| d__Bacteria;p__Actinobacteriota;c__Actinobacteria;o__Micrococcales;f__Intrasporangiaceae;__ | 0.001909 |
| d__Bacteria;p__Proteobacteria;c__Alphaproteobacteria;o__Caulobacterales;f__Caulobacteraceae;g__Brevundimonas | 0.005855 |
| d__Bacteria;p__Firmicutes;c__Clostridia;o__Clostridiales;f__Clostridiaceae;g__Clostridium_sensu_stricto_1 | 0.005633 |
| d__Bacteria;p__Firmicutes;c__Clostridia;o__Peptostreptococcales-Tissierellales;f__Anaerovoracaceae;g__Family_XIII_AD3011_group | 0.008409 |
| d__Bacteria;p__Firmicutes;c__Clostridia;o__Oscillospirales;f__Ruminococcaceae;__ | 0.01706 |
| d__Bacteria;p__Firmicutes;c__Clostridia;o__Oscillospirales;f__Ruminococcaceae;g__uncultured | 0.002051 |
| d__Bacteria;p__Bacteroidota;c__Bacteroidia;o__Bacteroidales;f__Rikenellaceae;g__Rikenellaceae_RC9_gut_group | 0.076169 |
| d__Bacteria;p__Firmicutes;c__Clostridia;o__Oscillospirales;f__UCG-010;g__UCG-010 | 0.014468 |
| d__Bacteria;p__Firmicutes;c__Clostridia;o__Lachnospirales;f__Lachnospiraceae;g__Lachnospiraceae_UCG-010 | 0.002076 |
| d__Bacteria;p__Acidobacteriota;c__Acidobacteriae;o__Acidobacteriales;f__Acidobacteriaceae__Subgroup_1_;g__uncultured | 0.000319 |
| d__Bacteria;p__Actinobacteriota;c__Actinobacteria;o__Micrococcales;f__Micrococcaceae;g__Pseudarthrobacter | 0.000564 |
| d__Bacteria;p__Firmicutes;c__Clostridia;o__Peptostreptococcales-Tissierellales;f__Anaerovoracaceae;g___Eubacterium__nodatum_group | 0.001322 |
| d__Bacteria;p__Firmicutes;c__Clostridia;o__Peptostreptococcales-Tissierellales;f__Anaerovoracaceae;g__Family_XIII_UCG-001 | 0.004686 |
| d__Bacteria;p__Firmicutes;c__Clostridia;o__Lachnospirales;f__Lachnospiraceae;g__Tuzzerella | 0.002417 |
| d__Bacteria;p__Actinobacteriota;c__Actinobacteria;o__Micrococcales;f__Intrasporangiaceae;g__Terrabacter | 0.001246 |
| d__Bacteria;p__Firmicutes;c__Clostridia;o__Peptostreptococcales-Tissierellales;f__Peptostreptococcaceae;g__Romboutsia | 0.001736 |
| d__Bacteria;p__Firmicutes;c__Clostridia;o__Lachnospirales;f__Lachnospiraceae;g__Lachnospiraceae_UCG-004 | 5.50E-05 |
| d__Bacteria;p__Firmicutes;c__Bacilli;o__Bacillales;f__Bacillaceae;__ | 5.80E-05 |
| d__Bacteria;p__Bacteroidota;c__Bacteroidia;o__Bacteroidales;f__Prevotellaceae;g__Alloprevotella | 0.001687 |
| d__Bacteria;p__Acidobacteriota;c__Acidobacteriae;o__Solibacterales;f__Solibacteraceae;g__Candidatus_Solibacter | 0.001346 |
| d__Bacteria;p__Actinobacteriota;c__Actinobacteria;o__Propionibacteriales;f__Nocardioidaceae;g__Nocardioides | 0.001511 |
| d__Bacteria;p__Firmicutes;c__Clostridia;o__Oscillospirales;f__Ruminococcaceae;g__Paludicola | 0.010261 |
| d__Bacteria;p__Firmicutes;c__Clostridia;o__Oscillospirales;f__Ruminococcaceae;g__Ruminococcaceae | 0.062493 |

**Supplement Table2.** FLI and WLI common features

| **Features** | **SUM_Abundance** | |
| --- | --- | --- |
| d__Bacteria;p__Firmicutes;c__Clostridia;o__Lachnospirales;f__Lachnospiraceae;g__Lachnospiraceae_FCS020_group | | 0.013846 |
| d__Bacteria;p__Firmicutes;c__Clostridia;o__Lachnospirales;f__Lachnospiraceae;__ | | 1.200607 |
| d__Bacteria;p__Firmicutes;c__Clostridia;o__Lachnospirales;f__Lachnospiraceae;g__Lachnospiraceae_NK4A136_group | | 0.349113 |
| d__Bacteria;p__Actinobacteriota;c__Coriobacteriia;o__Coriobacteriales;f__Eggerthellaceae;g__Enterorhabdus | | 0.015028 |
| d__Bacteria;p__Firmicutes;c__Clostridia;o__Oscillospirales;f__Ruminococcaceae;g__Incertae_Sedis | | 0.054215 |
| d__Bacteria;p__Bacteroidota;c__Bacteroidia;o__Bacteroidales;f__Muribaculaceae;g__Muribaculaceae | | 2.992421 |
| d__Bacteria;p__Firmicutes;c__Bacilli;__;__;__ | | 0.000958 |
| d__Bacteria;p__Bacteroidota;c__Bacteroidia;o__Bacteroidales;f__Rikenellaceae;g__Alistipes | | 0.813962 |
| d__Bacteria;p__Proteobacteria;c__Alphaproteobacteria;o__Sphingomonadales;f__Sphingomonadaceae;g__Sphingomonas | | 0.198879 |
| d__Bacteria;p__Firmicutes;c__Clostridia;o__Clostridia_vadinBB60_group;f__Clostridia_vadinBB60_group;g__Clostridia_vadinBB60_group | | 0.038795 |
| d__Bacteria;p__Firmicutes;c__Clostridia;o__Oscillospirales;f__Oscillospiraceae;g__uncultured | | 0.06392 |
| d__Bacteria;p__Proteobacteria;c__Alphaproteobacteria;o__Rhodospirillales;f__uncultured;g__uncultured | | 0.042572 |
| d__Bacteria;p__Firmicutes;c__Clostridia;o__Lachnospirales;f__Lachnospiraceae;g__GCA-900066575 | | 0.019527 |
| d__Bacteria;p__Firmicutes;c__Bacilli;o__Erysipelotrichales;f__Erysipelotrichaceae;g__uncultured | | 0.001751 |
| d__Bacteria;p__Firmicutes;c__Clostridia;o__Oscillospirales;f__Oscillospiraceae;g__Colidextribacter | | 0.02525 |
| d__Bacteria;p__Chloroflexi;c__Ktedonobacteria;o__C0119;f__C0119;g__C0119 | | 0.000302 |
| d__Bacteria;p__Actinobacteriota;c__Actinobacteria;o__Corynebacteriales;f__Corynebacteriaceae;g__Corynebacterium | | 0.007775 |
| d__Bacteria;p__Firmicutes;c__Clostridia;o__Lachnospirales;f__Lachnospiraceae;g__Lachnoclostridium | | 0.152041 |
| d__Bacteria;p__Firmicutes;c__Clostridia;o__Lachnospirales;f__Lachnospiraceae;g__Blautia | | 0.044743 |
| d__Bacteria;p__Firmicutes;c__Bacilli;o__Erysipelotrichales;f__Erysipelotrichaceae;g__Erysipelotrichaceae | | 0.01773 |
| d__Bacteria;p__Firmicutes;c__Clostridia;o__Clostridia_UCG-014;f__Clostridia_UCG-014;g__Clostridia_UCG-014 | | 0.596515 |
| d__Bacteria;p__Firmicutes;c__Clostridia;o__Oscillospirales;f__Oscillospiraceae;__ | | 0.25542 |
| d__Bacteria;p__Firmicutes;c__Clostridia;o__Christensenellales;f__Christensenellaceae;g__uncultured | | 0.001048 |
| d__Bacteria;p__Firmicutes;c__Clostridia;o__Clostridiales;f__Clostridiaceae;g__Candidatus_Arthromitus | | 0.023434 |
| d__Bacteria;p__Patescibacteria;c__Saccharimonadia;o__Saccharimonadales;f__Saccharimonadaceae;g__Candidatus_Saccharimonas | | 0.349514 |
| d__Bacteria;p__Actinobacteriota;c__Actinobacteria;o__Streptomycetales;f__Streptomycetaceae;g__Streptomyces | | 0.019321 |
| d__Bacteria;p__Bacteroidota;c__Bacteroidia;o__Bacteroidales;f__Bacteroidaceae;g__Bacteroides | | 3.216377 |
| d__Bacteria;p__Firmicutes;c__Clostridia;o__Lachnospirales;f__Lachnospiraceae;g__uncultured | | 0.00419 |
| d__Bacteria;p__Firmicutes;c__Clostridia;o__Oscillospirales;f__Oscillospiraceae;g__Oscillibacter | | 0.009678 |
| d__Bacteria;p__Bacteroidota;c__Bacteroidia;o__Bacteroidales;f__Marinifilaceae;g__Odoribacter | | 0.014581 |
| d__Bacteria;p__Firmicutes;c__Clostridia;o__Lachnospirales;f__Lachnospiraceae;g__Lachnospiraceae_UCG-006 | | 0.23237 |
| d__Bacteria;p__Desulfobacterota;c__Desulfovibrionia;o__Desulfovibrionales;f__Desulfovibrionaceae;g__uncultured | | 0.049634 |
| d__Bacteria;p__Firmicutes;c__Bacilli;o__Lactobacillales;f__Lactobacillaceae;g__Lactobacillus | | 0.001299 |
| d__Bacteria;p__Firmicutes;c__Clostridia;o__Oscillospirales;f__Oscillospiraceae;g__NK4A214_group | | 0.004639 |
| d__Bacteria;p__Bacteroidota;c__Bacteroidia;o__Chitinophagales;f__Chitinophagaceae;g__Flavisolibacter | | 0.003746 |
| d__Bacteria;p__Firmicutes;c__Clostridia;o__Lachnospirales;f__Lachnospiraceae;g__Acetatifactor | | 0.004919 |
| d__Bacteria;p__Firmicutes;c__Clostridia;o__Oscillospirales;f___Eubacterium__coprostanoligenes_group;g___Eubacterium__coprostanoligenes_group | | 0.006653 |
| d__Bacteria;p__Firmicutes;c__Clostridia;o__Lachnospirales;f__Lachnospiraceae;g__Marvinbryantia | | 0.010172 |
| d__Bacteria;p__Acidobacteriota;c__Acidobacteriae;o__Bryobacterales;f__Bryobacteraceae;g__Bryobacter | | 0.001078 |
| d__Bacteria;p__Chloroflexi;c__KD4-96;o__KD4-96;f__KD4-96;g__KD4-96 | | 0.000555 |
| d__Bacteria;p__Firmicutes;c__Bacilli;o__Lactobacillales;__;__ | | 0.000109 |
| d__Bacteria;p__Firmicutes;c__Clostridia;o__Lachnospirales;f__Lachnospiraceae;g__ASF356 | | 0.002002 |
| d__Bacteria;p__Proteobacteria;c__Alphaproteobacteria;o__Rhizobiales;f__Rhizobiaceae;g__Ochrobactrum | | 0.001233 |
| d__Bacteria;p__Bacteroidota;c__Bacteroidia;o__Bacteroidales;f__Tannerellaceae;g__Parabacteroides | | 0.035677 |
| d__Bacteria;p__Firmicutes;c__Bacilli;o__Bacillales;f__Bacillaceae;g__Bacillus | | 0.000378 |
| d__Bacteria;p__Bacteroidota;c__Bacteroidia;o__Bacteroidales;f__Prevotellaceae;g__Prevotellaceae_UCG-001 | | 0.001898 |
| d__Bacteria;p__Bacteroidota;c__Bacteroidia;o__Chitinophagales;f__Chitinophagaceae;g__Chitinophagaceae | | 0.000609 |
| d__Bacteria;p__Firmicutes;c__Clostridia;o__Oscillospirales;f__Butyricicoccaceae;g__Butyricicoccus | | 0.018332 |
| d__Bacteria;p__Actinobacteriota;c__Actinobacteria;o__Corynebacteriales;f__Nocardiaceae;g__Nocardia | | 0.001387 |
| d__Bacteria;p__Gemmatimonadota;c__Gemmatimonadetes;o__Gemmatimonadales;f__Gemmatimonadaceae;g__Gemmatimonas | | 0.000454 |
| d__Bacteria;p__Actinobacteriota;c__Actinobacteria;o__Bifidobacteriales;f__Bifidobacteriaceae;g__Bifidobacterium | | 0.059509 |
| d__Bacteria;p__Firmicutes;c__Bacilli;o__Erysipelotrichales;f__Erysipelatoclostridiaceae;g__Erysipelatoclostridium | | 0.018141 |
| d__Bacteria;p__Firmicutes;c__Clostridia;o__Peptococcales;f__Peptococcaceae;g__uncultured | | 0.001134 |
| d__Bacteria;p__Actinobacteriota;c__Actinobacteria;o__Micrococcales;f__Intrasporangiaceae;__ | | 0.001909 |
| d__Bacteria;p__Proteobacteria;c__Alphaproteobacteria;o__Caulobacterales;f__Caulobacteraceae;g__Brevundimonas | | 0.005855 |
| d__Bacteria;p__Firmicutes;c__Clostridia;o__Clostridiales;f__Clostridiaceae;g__Clostridium_sensu_stricto_1 | | 0.005633 |
| d__Bacteria;p__Firmicutes;c__Clostridia;o__Peptostreptococcales-Tissierellales;f__Anaerovoracaceae;g__Family_XIII_AD3011_group | | 0.008409 |
| d__Bacteria;p__Firmicutes;c__Clostridia;o__Oscillospirales;f__Ruminococcaceae;__ | | 0.01706 |
| d__Bacteria;p__Firmicutes;c__Clostridia;o__Oscillospirales;f__Ruminococcaceae;g__uncultured | | 0.002051 |
| d__Bacteria;p__Bacteroidota;c__Bacteroidia;o__Bacteroidales;f__Rikenellaceae;g__Rikenellaceae_RC9_gut_group | | 0.076169 |
| d__Bacteria;p__Firmicutes;c__Clostridia;o__Oscillospirales;f__UCG-010;g__UCG-010 | | 0.014468 |
| d__Bacteria;p__Firmicutes;c__Clostridia;o__Lachnospirales;f__Lachnospiraceae;g__Lachnospiraceae_UCG-010 | | 0.002076 |
| d__Bacteria;p__Acidobacteriota;c__Acidobacteriae;o__Acidobacteriales;f__Acidobacteriaceae__Subgroup_1_;g__uncultured | | 0.000319 |
| d__Bacteria;p__Actinobacteriota;c__Actinobacteria;o__Micrococcales;f__Micrococcaceae;g__Pseudarthrobacter | | 0.000564 |
| d__Bacteria;p__Firmicutes;c__Clostridia;o__Peptostreptococcales-Tissierellales;f__Anaerovoracaceae;g___Eubacterium__nodatum_group | | 0.001322 |
| d__Bacteria;p__Firmicutes;c__Clostridia;o__Peptostreptococcales-Tissierellales;f__Anaerovoracaceae;g__Family_XIII_UCG-001 | | 0.004686 |
| d__Bacteria;p__Firmicutes;c__Clostridia;o__Lachnospirales;f__Lachnospiraceae;g__Tuzzerella | | 0.002417 |
| d__Bacteria;p__Actinobacteriota;c__Actinobacteria;o__Micrococcales;f__Intrasporangiaceae;g__Terrabacter | | 0.001246 |
| d__Bacteria;p__Firmicutes;c__Clostridia;o__Peptostreptococcales-Tissierellales;f__Peptostreptococcaceae;g__Romboutsia | | 0.001736 |
| d__Bacteria;p__Firmicutes;c__Clostridia;o__Lachnospirales;f__Lachnospiraceae;g__Lachnospiraceae_UCG-004 | | 5.50E-05 |
| d__Bacteria;p__Firmicutes;c__Bacilli;o__Bacillales;f__Bacillaceae;__ | | 5.80E-05 |
| d__Bacteria;p__Bacteroidota;c__Bacteroidia;o__Bacteroidales;f__Prevotellaceae;g__Alloprevotella | | 0.001687 |
| d__Bacteria;p__Acidobacteriota;c__Acidobacteriae;o__Solibacterales;f__Solibacteraceae;g__Candidatus_Solibacter | | 0.001346 |
| d__Bacteria;p__Actinobacteriota;c__Actinobacteria;o__Propionibacteriales;f__Nocardioidaceae;g__Nocardioides | | 0.001511 |
| d__Bacteria;p__Firmicutes;c__Clostridia;o__Oscillospirales;f__Ruminococcaceae;g__Paludicola | | 0.010261 |
| d__Bacteria;p__Firmicutes;c__Clostridia;o__Oscillospirales;f__Ruminococcaceae;g__Ruminococcaceae | | 0.062493 |

**Supplement Table3.** WIMT and FMT common features

| **Features** | **SUM_Abundance** |
| --- | --- |
| d__Bacteria;p__Firmicutes;c__Clostridia;o__Lachnospirales;f__Lachnospiraceae;__ | 0.163177 |
| d__Bacteria;p__Firmicutes;c__Clostridia;o__Lachnospirales;f__Lachnospiraceae;g__Lachnospiraceae_NK4A136_group | 0.056707 |
| d__Bacteria;p__Actinobacteriota;c__Coriobacteriia;o__Coriobacteriales;f__Eggerthellaceae;g__Enterorhabdus | 0.009559 |
| d__Bacteria;p__Firmicutes;c__Clostridia;o__Oscillospirales;f__Ruminococcaceae;g__Incertae_Sedis | 0.026983 |
| d__Bacteria;p__Bacteroidota;c__Bacteroidia;o__Bacteroidales;f__Muribaculaceae;g__Muribaculaceae | 5.047178 |
| d__Bacteria;p__Firmicutes;c__Bacilli;__;__;__ | 0.000994 |
| d__Bacteria;p__Bacteroidota;c__Bacteroidia;o__Bacteroidales;f__Rikenellaceae;g__Alistipes | 0.410897 |
| d__Bacteria;p__Proteobacteria;c__Alphaproteobacteria;o__Sphingomonadales;f__Sphingomonadaceae;g__Sphingomonas | 0.001713 |
| d__Bacteria;p__Firmicutes;c__Clostridia;o__Clostridia_vadinBB60_group;f__Clostridia_vadinBB60_group;g__Clostridia_vadinBB60_group | 0.069213 |
| d__Bacteria;p__Firmicutes;c__Clostridia;o__Oscillospirales;f__Oscillospiraceae;g__uncultured | 0.039709 |
| d__Bacteria;p__Proteobacteria;c__Alphaproteobacteria;o__Rhodospirillales;f__uncultured;g__uncultured | 0.063146 |
| d__Bacteria;p__Firmicutes;c__Clostridia;o__Lachnospirales;f__Lachnospiraceae;g__GCA-900066575 | 0.003602 |
| d__Bacteria;p__Firmicutes;c__Bacilli;o__Erysipelotrichales;f__Erysipelotrichaceae;g__uncultured | 0.001567 |
| d__Bacteria;p__Firmicutes;c__Clostridia;o__Oscillospirales;f__Oscillospiraceae;g__Colidextribacter | 0.00337 |
| d__Bacteria;p__Actinobacteriota;c__Actinobacteria;o__Corynebacteriales;f__Corynebacteriaceae;g__Corynebacterium | 0.018379 |
| d__Bacteria;p__Firmicutes;c__Clostridia;o__Lachnospirales;f__Lachnospiraceae;g__Lachnoclostridium | 0.041618 |
| d__Bacteria;p__Verrucomicrobiota;c__Verrucomicrobiae;o__Verrucomicrobiales;f__Akkermansiaceae;g__Akkermansia | 0.002808 |
| d__Bacteria;p__Firmicutes;c__Clostridia;o__Lachnospirales;f__Lachnospiraceae;g__Blautia | 0.032923 |
| d__Bacteria;p__Firmicutes;c__Bacilli;o__Erysipelotrichales;f__Erysipelotrichaceae;g__Erysipelotrichaceae | 0.004554 |
| d__Bacteria;p__Firmicutes;c__Clostridia;o__Clostridia_UCG-014;f__Clostridia_UCG-014;g__Clostridia_UCG-014 | 0.673645 |
| d__Bacteria;p__Firmicutes;c__Clostridia;o__Oscillospirales;f__Oscillospiraceae;__ | 0.106969 |
| d__Bacteria;p__Firmicutes;c__Clostridia;o__Christensenellales;f__Christensenellaceae;g__uncultured | 0.000182 |
| d__Bacteria;p__Firmicutes;c__Clostridia;o__Clostridiales;f__Clostridiaceae;g__Candidatus_Arthromitus | 0.00191 |
| d__Bacteria;p__Patescibacteria;c__Saccharimonadia;o__Saccharimonadales;f__Saccharimonadaceae;g__Candidatus_Saccharimonas | 0.344786 |
| d__Bacteria;p__Bacteroidota;c__Bacteroidia;o__Bacteroidales;f__Bacteroidaceae;g__Bacteroides | 3.165247 |
| d__Bacteria;p__Firmicutes;c__Clostridia;o__Lachnospirales;f__Lachnospiraceae;g__uncultured | 0.001018 |
| d__Bacteria;p__Firmicutes;c__Clostridia;o__Oscillospirales;f__Oscillospiraceae;g__Oscillibacter | 0.001998 |
| d__Bacteria;p__Firmicutes;c__Clostridia;o__Oscillospirales;__;__ | 0.00032 |
| d__Bacteria;p__Bacteroidota;c__Bacteroidia;o__Bacteroidales;f__Marinifilaceae;g__Odoribacter | 0.021882 |
| d__Bacteria;p__Firmicutes;c__Clostridia;o__Lachnospirales;f__Lachnospiraceae;g__Lachnospiraceae_UCG-006 | 0.029873 |
| d__Bacteria;p__Desulfobacterota;c__Desulfovibrionia;o__Desulfovibrionales;f__Desulfovibrionaceae;g__uncultured | 0.01383 |
| d__Bacteria;p__Actinobacteriota;c__Actinobacteria;o__Micrococcales;f__Dermabacteraceae;g__Brachybacterium | 0.000621 |
| d__Bacteria;p__Firmicutes;c__Clostridia;o__Lachnospirales;f__Lachnospiraceae;g__Acetatifactor | 0.000618 |
| d__Bacteria;p__Firmicutes;c__Clostridia;o__Oscillospirales;f___Eubacterium__coprostanoligenes_group;g___Eubacterium__coprostanoligenes_group | 0.012269 |
| d__Bacteria;p__Firmicutes;c__Clostridia;o__Lachnospirales;f__Lachnospiraceae;g__Marvinbryantia | 0.005409 |
| d__Bacteria;p__Proteobacteria;c__Alphaproteobacteria;o__Rhizobiales;f__Rhizobiaceae;g__Allorhizobium-Neorhizobium-Pararhizobium-Rhizobium | 0.000747 |
| d__Bacteria;p__Bacteroidota;c__Bacteroidia;o__Bacteroidales;f__Tannerellaceae;g__Parabacteroides | 0.064456 |
| d__Bacteria;p__Firmicutes;c__Bacilli;o__Bacillales;f__Bacillaceae;g__Bacillus | 0.000921 |
| d__Bacteria;p__Bacteroidota;c__Bacteroidia;o__Bacteroidales;f__Prevotellaceae;g__Prevotellaceae_UCG-001 | 0.00896 |
| d__Bacteria;p__Firmicutes;c__Clostridia;o__Oscillospirales;f__Butyricicoccaceae;g__Butyricicoccus | 0.001318 |
| d__Bacteria;p__Firmicutes;c__Clostridia;o__Oscillospirales;f__Ruminococcaceae;g__Harryflintia | 0.002732 |
| d__Bacteria;p__Desulfobacterota;c__Desulfovibrionia;o__Desulfovibrionales;f__Desulfovibrionaceae;__ | 4.66E-05 |
| d__Bacteria;p__Actinobacteriota;c__Actinobacteria;o__Bifidobacteriales;f__Bifidobacteriaceae;g__Bifidobacterium | 0.149288 |
| d__Bacteria;p__Firmicutes;c__Clostridia;o__Lachnospirales;f__Lachnospiraceae;g__Hungatella | 0.002127 |
| d__Bacteria;p__Firmicutes;c__Bacilli;o__Erysipelotrichales;f__Erysipelatoclostridiaceae;g__Erysipelatoclostridium | 0.02276 |
| d__Bacteria;p__Firmicutes;c__Clostridia;o__Peptococcales;f__Peptococcaceae;g__uncultured | 0.000364 |
| d__Bacteria;p__Firmicutes;c__Clostridia;o__Peptostreptococcales-Tissierellales;f__Anaerovoracaceae;g__Family_XIII_AD3011_group | 0.002036 |
| d__Bacteria;p__Firmicutes;c__Clostridia;o__Oscillospirales;f__Ruminococcaceae;__ | 0.00668 |
| d__Bacteria;p__Firmicutes;c__Clostridia;o__Oscillospirales;f__Ruminococcaceae;g__uncultured | 0.001405 |
| d__Bacteria;p__Bacteroidota;c__Bacteroidia;o__Bacteroidales;f__Rikenellaceae;g__Rikenellaceae_RC9_gut_group | 0.057383 |
| d__Bacteria;p__Firmicutes;c__Clostridia;o__Oscillospirales;f__UCG-010;g__UCG-010 | 0.035537 |
| d__Bacteria;p__Bacteroidota;c__Bacteroidia;o__Bacteroidales;f__Rikenellaceae;g__Rikenella | 0.003438 |
| d__Bacteria;p__Actinobacteriota;c__Coriobacteriia;o__Coriobacteriales;f__Eggerthellaceae;g__uncultured | 0.000902 |
| d__Bacteria;p__Firmicutes;c__Clostridia;o__Peptostreptococcales-Tissierellales;f__Anaerovoracaceae;g___Eubacterium__nodatum_group | 0.000397 |
| d__Bacteria;p__Firmicutes;c__Clostridia;o__Lachnospirales;f__Lachnospiraceae;g__Tuzzerella | 0.000444 |
| d__Bacteria;p__Actinobacteriota;c__Actinobacteria;o__Micrococcales;f__Brevibacteriaceae;g__Brevibacterium | 0.001104 |
| d__Bacteria;p__Firmicutes;c__Bacilli;o__Erysipelotrichales;f__Erysipelotrichaceae;g___Clostridium__innocuum_group | 0.002991 |
| d__Bacteria;p__Bacteroidota;c__Bacteroidia;o__Bacteroidales;f__Prevotellaceae;g__Alloprevotella | 0.005215 |
| d__Bacteria;p__Firmicutes;c__Clostridia;o__Oscillospirales;f__Ruminococcaceae;g__Paludicola | 0.004819 |
| d__Bacteria;p__Firmicutes;c__Clostridia;o__Oscillospirales;f__Ruminococcaceae;g__Ruminococcaceae | 0.08378 |

**Supplement Table4**. Enrichment analysis of fecal microbial dominant pathways in the WIMT group and the FMT group

| ID | enriched | P value |
| --- | --- | --- |
| ARGSYN-PWY | FMT | 0.041126 |
| ARO-PWY | FMT | 0.025974 |
| COBALSYN-PWY | FMT | 0.041126 |
| COMPLETE-ARO-PWY | FMT | 0.025974 |
| DAPLYSINESYN-PWY | FMT | 0.041126 |
| PWY-5104 | FMT | 0.008658 |
| PWY-5509 | FMT | 0.041126 |
| PWY-6163 | FMT | 0.041126 |
| PWY-6269 | FMT | 0.041126 |
| PWY-6478 | FMT | 0.008658 |
| PWY-7400 | FMT | 0.041126 |
| PWY0-1297 | FMT | 0.041126 |
| PWY-5180 | WIMT | 0.009622 |
| PWY-5415 | WIMT | 0.009622 |
| PWY0-845 | WIMT | 0.008658 |
| PYRIDOXSYN-PWY | WIMT | 0.015152 |
| RHAMCAT-PWY | WIMT | 0.025974 |
| PWY-6612 | WIMT | 0.041126 |
| PWY-7237 | WIMT | 0.008658 |
| DENOVOPURINE2-PWY | WIMT | 0.041126 |
| DTDPRHAMSYN-PWY | WIMT | 0.025974 |
| HISDEG-PWY | WIMT | 0.015152 |
| PROTOCATECHUATE-ORTHO-CLEAVAGE-PWY | WIMT | 0.02948 |
